# Kidney dosimetry in 777 patients during ^177^Lu-DOTATATE therapy: aspects on extrapolations and measurement time points

**DOI:** 10.1186/s40658-020-00339-2

**Published:** 2020-12-09

**Authors:** Mattias Sandström, Nanette Freedman, Katarzyna Fröss-Baron, Tanweera Kahn, Anders Sundin

**Affiliations:** 1 Section of Nuclear Medicine and PET, Department of Surgical Sciences, Uppsala University, Uppsala, Sweden; 2 Section of Medical Physics, Department of Immunology, Genetics and Pathology, Uppsala University, 751 85 Uppsala, Sweden; 3 Institute of Nuclear Medicine, Tel Aviv Sourasky Medical Center, Tel Aviv, Israel; 4 Division of Endocrine Oncology, Department of Medical Sciences, Uppsala University Hospital, Uppsala, Sweden; 5 Section of Oncology, Department of Immunology, Genetics and Pathology, Uppsala University, Uppsala, Sweden

**Keywords:** Extrapolation, Simplifications, Dosimetry, Kidney, ^177^Lu-DOTATATE, Neuroendocrine tumors

## Abstract

**Purpose:**

Fractionated peptide receptor radionuclide therapy (PRRT) with ^177^Lu-DOTATATE is increasingly applied as an effective treatment for patients with disseminated neuroendocrine tumors. In parallel to dose planning before external beam radiation therapy, dosimetry is also needed to optimize PRRT to the individual patient. Accordingly, absorbed doses to organs at risk need to be calculated during PRRT, based on serial measurements of radioactivity distribution utilizing SPECT/CT. The dosimetry should be based on as few measurements as possible, while still retaining reliable results. The main aim of the present work was to calculate the fractional contribution of the extrapolations of the curve fits for the absorbed dose calculations to the kidneys. The secondary aim was to study agreement between absorbed dose (AD) and the effective half-life (*t*_eff_) for the kidneys, estimated by means of measurements at one or two time points, in comparison to our current method employing three time points.

**Methods:**

In 777 patients with disseminated neuroendocrine tumors undergoing PRRT, SPECT/CT over the abdomen was acquired at 1, 4, and 7 days after ^177^Lu-DOTATATE infusion. The absorbed dose to the kidneys was calculated from SPECT/CT radioactivity distribution data, and the *t*_eff_ and fractional contributions of the extrapolations were estimated, utilizing data from one, two, and three time points, respectively.

**Results:**

The fractional contributions from extrapolations before day 1 measurement and after day 7 measurement were approximately 26% and 11%, respectively. The mean differences in absorbed dose, based on one, two, and three time points were small, but with high method dependence for individual patients. The differences in estimated *t*_eff_ were small when it was based on measurements at days 1 and 7, but high for days 1 and 4 time points.

**Conclusion:**

When assessing simplifications of methods for calculation of the absorbed dose to the kidneys, it was of the uttermost importance to incorporate the fractional contribution for the extrapolations included in the reference method. Measurements at an early and a late time point were found most important. An intermediate measurement contributes with an idea of the goodness of the fit.

## Introduction

During the last decade, peptide receptor radionuclide therapy (PRRT) with ^177^Lu-DOTA-D-Phe1-Tyr3-octreotate (^177^Lu-DOTATATE) has evolved as an effective treatment option for patients with disseminated somatostatin receptor positive neuroendocrine tumors (NET) [1–8]. In external beam radiation therapy, dose planning ensures that the target receives a predefined absorbed dose without inducing severe side effects on surrounding tissues. It is also well established that a higher absorbed dose on a tissue introduces a larger damage on the tissue, even if the response is not linear and with possible thresholds both for normal organ and tumor response. There is no evidence that this would not be advantageous also in the setting of targeted radiotherapy, where the radiation originates from a radiotracer. On the contrary, accumulating scientific evidence supports such a dose-response relationship in PRRT. For example, Pauwels et al. [9] showed a correlation between the absorbed dose and tumor shrinkage for PRRT with ^90^Y-DOTATOC, and for treatment with ^177^Lu-DOTATATE, Ilan et al. [10] showed a good correlation between absorbed dose and shrinkage of pancreatic neuroendocrine tumors, and Jahn et al. [11] showed a significant correlation between the injected activity and tumor shrinkage for small intestinal neuroendocrine tumors.

The current standard in ^177^Lu-DOTATATE therapy, based on the original Rotterdam protocol, is to administer four 7.4 GBq cycles (29.6 GBq in total), which is considered safe for the organs at risk in the majority of patients. Moreover, in recent ^177^Lu-DOTATATE trials, only occasional patients have experienced significant renal toxicity (grades 3–4) [12]. This may be illustrated by a dosimetry-tailored dose escalation study in 200 patients receiving 22.2–74 GBq [8] and a study in 74 patients receiving 14.8–37.8 GBq [13], in both of which only one patient showed renal toxicity grades 3–4. The administered activity to the individual patient could therefore most probably be increased in order to increase the absorbed dose to the tumors. This is also supported by the results in 51 patients who received median 5 (range 3–7) cycles of 7.4 GBq ^177^Lu-DOTATATE up to 27 Gy biological effective dose (BED) to the kidneys and in 5 patients in whom this was further increased to 40 Gy BED, allowing for administering median 7 (range 5–8) cycles [14]. GFR decreased in most patients, but no grades 3–4 renal toxicity were observed in this study. Similarly, this is supported by the results in a quite recent Canadian study by Del Prete et al. [15]. Neither salvage treatment, administering two additional PRRT cycles up to 60.5 GBq cumulative activity, shown any increase in kidney and bone marrow-related side effects [16]. However, there are no published data for the patient outcome and radiation-related side effects for dosimetry-guided PRRT, allowing for > 4 initial cycles, in comparison to a treatment-re-treatment regimen. Neither is it known how the PRRT protocol is best adapted to benefit the outcome for the different types of NETs. It is obvious that the once established 23 Gy upper limit for absorbed dose to the kidneys is too low, and in order to establish the true upper limit, dosimetry-guided PRRT is needed. By taking advantage of quantitative imaging, this would not only allow for tailoring the thresholds for normal organs, but also to perform tumor dosimetry and optimize the absorbed dose to tumor tissue, and thereby provides possibilities to individualize the subsequent treatment cycles.

Although the kidney toxicity with ^177^Lu-DOTATATE is less than for ^90^Y-labeled preparations [1, 17, 18], as pointed out above, the maximum tolerable absorbed dose remains to be defined [4, 19], and a reliable and preferably not too extensive dosimetry is required for the individual patient. For PRRT with ^177^Lu-DOTATATE, calculation of the absorbed dose is based on the activity distribution and kinetics over time for the relevant organs and tissues, generally obtained by gamma camera measurements repeated over time. To calculate the entire time course of activity distribution from injection time out to infinity, interpolations between the measurements and extrapolations before the first and after the last measurement points are required. To avoid errors in time-integrated activity and absorbed dose keeping these errors as small as possible, the area under the curve of the extrapolations should be as small as possible and preferably include less than 20% of the decays in the volume [20]. This is important from two perspectives. The first is that large extrapolations of a constant function in a curve fit increases the uncertainty. The second is that we cannot presume that the biological conditions, affecting the biodistribution, are the same over time.

Because of logistical and financial reasons and for patient comfort, estimation of absorbed doses should be performed with as few measurements as possible, while still achieving reliable results of the absorbed dose calculations. A clinically applicable and robust dosimetry protocol for solid organs, based on 3D imaging by SPECT, has been developed and applied in our center since 2005 [21–23].

The main aim of the present work was to calculate the fractional contributions of the extrapolations of the curve fits for the absorbed dose calculations to the kidneys. The secondary aim was to study how well kidney-absorbed dose and effective half-life (*t*_eff_) estimations, using methods based on measurements at one or two time points, agree with the current method employing three time points.

## Materials and methods

### Patients

In this retrospective study, 777 patients (333 females and 444 males) with metastatic somatostatin receptor-positive neuroendocrine tumors treated with ^177^Lu-DOTATATE were included, and all of them met previously described inclusion criteria [21]. Dosimetry on these patients was performed during the years 2006 to 2019. Both the left and the right kidneys were included in the analysis, with the exception of the right kidney in 12 patients and the left kidney in 11 patients, in whom the kidneys had been resected or had very impaired function.

^177^LuCl_3_ was purchased from IDB Radiopharmacy bv, Baarle-Nassau, The Netherlands, and DOTATATE was a generous gift from Erasmus Medical Centre, Rotterdam, The Netherlands.

### Compliance with ethical standards

The study received no external funding, and all authors declare no conflict of interest.

Since September 2010, all patients were included into a prospective study (EudraCT no. 2009-012260-14) approved by the Regional Ethical Review Board in Uppsala. Before that, from 2005, the patients were admitted on a single-patient basis for compassionate use with individual permission from the Swedish Medical Products Agency. All patients gave their written informed consent before study inclusion.

### Image acquisition

All 777 patients underwent SPECT/CT of the abdomen 1, 4, and 7 days after administration of the first cycle of 7.4 GBq ^177^Lu-DOTATATE. For the first 69 patients, imaging was performed on a Hawkeye Millennium VG (GE Healthcare) dual-head camera equipped with 5/8” NaI(Tl) crystals and MEGP (medium energy general purpose) collimators. A 20% energy window around the 2 dominant γ-ray energies of ^177^Lu, 113.0 and 208.4 keV, was applied. SPECT/CT, applying 60 frames with a 60-s exposure time per frame (total acquisition time for SPECT is then 30 min), was performed over the upper abdomen including organs at risk (kidneys, liver, and spleen). In the next 400 patients, imaging was performed on an Infinia (International General Electric, General Electric Medical Systems, Haifa, Israel) dual-headed gamma camera with 3/8” NaI(Tl)-crystals equipped with MEGP collimators. The measurements employed a 20% energy window around the dominant 208.4 keV gamma ray energy of ^177^Lu. SPECT/CT of the upper abdomen included the organs at risk (kidneys, liver, and spleen), applying 120 frames with a 30-s exposure time per frame. In the last 308 patients, SPECT/CT was performed on a Discovery 670 PRO (International General Electric, General Electric Medical Systems, Haifa, Israel) dual-headed gamma camera with 3/8” NaI(Tl)-crystals equipped with MEGP collimators with the same settings as for the Infinia. For reconstruction, the ordered subset expectation maximization (OSEM) algorithm included in the Xeleris 3.0 workstation (International General Electric, General Electric Medical Systems, Haifa, Israel) was used with previously determined default settings (iterative reconstruction with eight subsets and four iterations followed by a Hann filtering with a cutoff of 0.85). In all the systems above, the images were attenuation corrected with the concomitantly acquired CT-based attenuation map but were not corrected for scatter, collimator/response, or PVE. The small VOI method is sensitive to artefacts, and because of the Gibbs artefacts, no collimator response/resolution recovery was included. Scatter correction was also omitted since the only available methods for us are the triple or dual energy window methods, which for us have generated more problems than they have solved.

### Absorbed dose calculations

All volumes of interests (VOIs) were defined using in-house-developed software within the Hermes platform on a Hermes HNAC workstation with the Gold 2.9 software (HERMES, Stockholm, Sweden).

In the SPECT images, small spherical volumes of interests (VOIs; 4 ml) were placed in both kidneys to include the renal cortex as described previously [21]. Activity concentrations were determined for each time point (1, 4, and 7 days after ^177^Lu-administration), and time-integrated activity concentration was calculated as the area under the curve of a single exponential fit (from infusion start to infinity) to the time-activity concentration curve (*A*(*t*)).

In the MIRD 21 pamphlet [24], the mean absorbed dose *D*(*r*_T_,*T*_D_) to a target structure *r*_T_ in the time period from time 0 to time *T*_D_ is defined as:

1 $$ D\left({r}_{\mathrm{T}},{T}_{\mathrm{D}}\right)=\sum \limits_{r_{\mathrm{s}}}{\int}_0^{T_{\mathrm{D}}}A\left({r}_{\mathrm{S}},t\right)S\left({r}_{\mathrm{T}}\leftarrow {r}_{\mathrm{S}},t\right) dt $$

where *A*(*r*_S_,*t*) is the activity of the radiopharmaceutical in source tissue *r*_S_ at time *t*, and *S*(*r*_T_ ← *r*_S_,*t*) is the radionuclide-specific quantity representing the mean absorbed dose rate to target tissue *r*_T_ at time *t* after administration per unit activity present in source tissue *r*_S_.

It has been shown [25] that the absorbed dose from surrounding organs to kidneys in therapy with ^177^Lu-DOTATATE does not add much to the absorbed dose. This general formula can be rewritten to only include the absorbed dose originating from the target structure itself:

2 $$ D\left({r}_{\mathrm{T}},{T}_{\mathrm{D}}\right)={\int}_0^{T_{\mathrm{D}}}A\left({r}_{\mathrm{T}},t\right)S\left({r}_{\mathrm{T}}\leftarrow {r}_{\mathrm{T}},t\right) dt $$

To simplify further, Eq. 2 can be rewritten again to work with concentrations instead.

3 $$ D\left({r}_{\mathrm{T}},{T}_{\mathrm{D}}\right)={\int}_0^{T_{\mathrm{D}}}C\left({r}_{\mathrm{T}},t\right)\ast \mathrm{ACDF}\left({r}_{\mathrm{T}}\leftarrow {r}_{\mathrm{T}},t\right) dt $$

where *C*(*r*_S_,*t*) is the activity concentration of the radiopharmaceutical in target tissue *r*_S_ at time *t*, and ACDF(*r*_T_ ← *r*_T_,*t*) (activity concentration dose factor) is the radionuclide-specific quantity representing the mean absorbed dose rate to target tissue *r*_T_ at time *t* after administration per unit activity concentration present in target tissue *r*_T_. ACDF is a multiplication of S-factor with the volume for the S-factor. The ACDF does not change much with the volume, and using dose factors (DF) from the spherical model in OLINDA [26] gives an ACDF of 86.0 mGy*g/MBq*h for a 100-g sphere and 86.7 mGy*g/MBq*h for a 300-g sphere leading to a difference of less than 1%.

Calculating the time-integrated activity concentration ($\overset{\sim }{C}$) from time of administration to infinity and assuming a density of 1 means that the final equation for calculation of the absorbed dose to the kidneys ends up with a simple multiplication:

4 $$ {D}_{\mathrm{Kidney}}=\overset{\sim }{C}\ast {\mathrm{ACDF}}_{\mathrm{Kidney}\leftarrow \mathrm{Kidney}} $$

This procedure has previously been described in more detail in the following references [21, 23].

### Fractional contributions

For the absorbed dose to the kidneys, several fractional contributions (*f*) (Eq. 5) were calculated for each therapy cycle of 7.4 GBq.

5 $$ f=\left(\frac{\int_{t_1}^{t_2}C(t) dt}{\int_{t_s}^{t_e}C(t) dt}\right) $$

In this equation, the fractional contribution (*f*) is defined as the area under the curve of the expression (*C*(*t*)) between the time of the first measurement point (*t*_1_) and the time of the last measurement point (*t*_2_) divided by the total area under the curve from the start time (*t*_s_) to the end time (*t*_e_). The expression (*C*(*t*)) is in this case the single exponential fit to the measurements of radioactivity concentration in the kidneys.

**Fig. 1:**
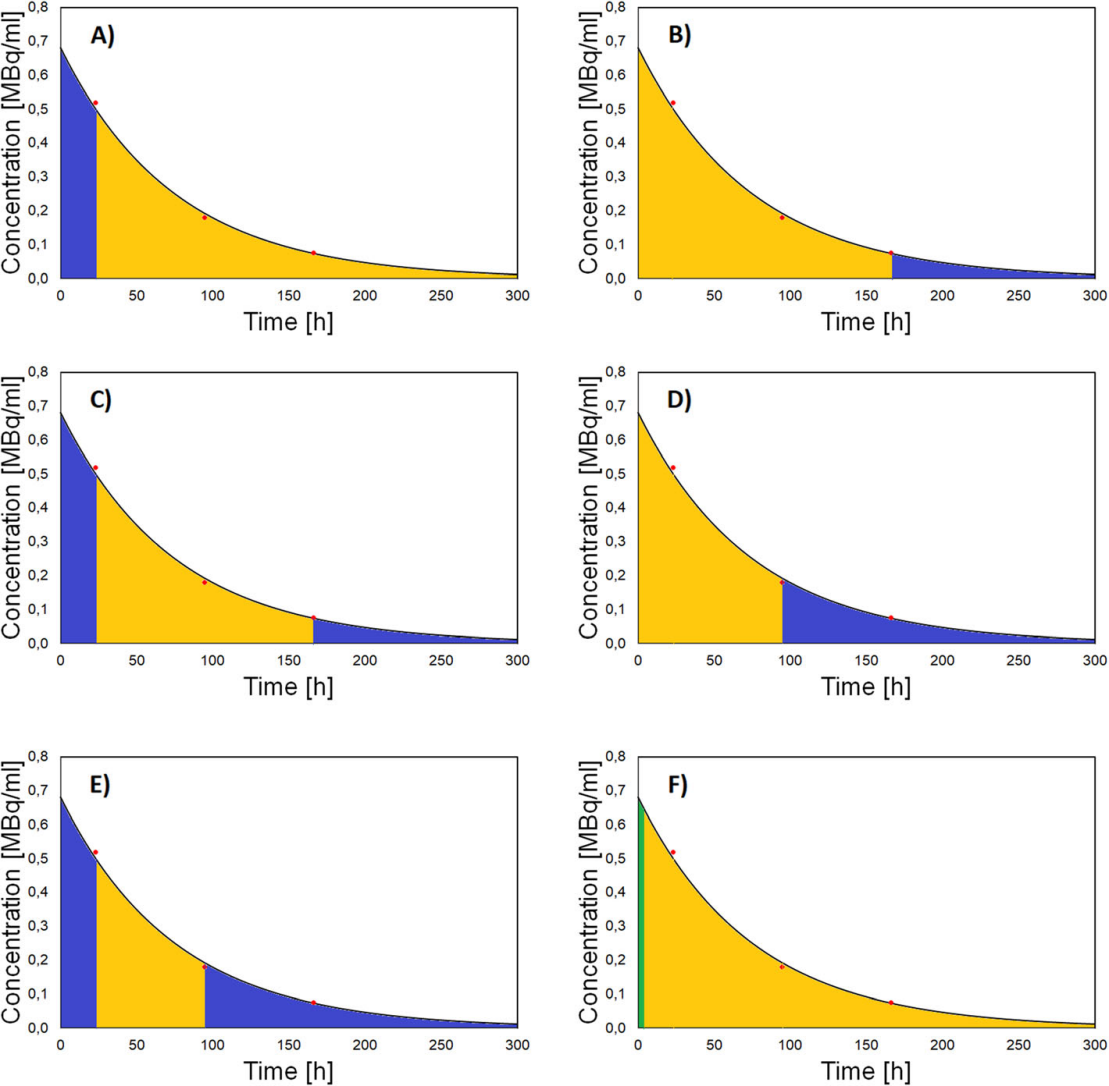
Examples of fractional contributions on a typical curve for the kidney for **a** (fc_0-24_) the extrapolated portion of the curve from time = 0 to the measurement at day 1, **b** (fc_168-∞_) the portion of the curve after the day 7 measurement (to infinity), **c** (fc_0-24 + 168-∞_) the sum of fc_0-24_ and fc_168-∞_, **d** (fc_96-∞_) the portion of the curve after the day 4 measurement (to infinity), **e** (fc_0-24 + 96-∞_) the sum of fc_0-24_ and fc_96-∞_, and (fc_0-8_) the fractional contribution of the first 8 h

The following fractional contributions were calculated on the single exponential curve fit on days 1, 4, and 7 measurements for the right and left kidney: (fc_0-24_) the extrapolated portion of the curve from time = 0 (start of ^177^Lu-DOTATATE infusion) to the measurement at day 1, (fc_168-∞_) the portion of the curve after day 7 measurement (to infinity), (fc_0-24+168-∞_) the sum of fc_0-24_ and fc_168-∞_, (fc_96-∞_) the portion of the curve after day 4 measurement (to infinity), and (fc_0-24+96-∞_) the sum of fc_0-24_ and fc_96-∞_; each of these extrapolations were calculated as a fraction of the total area under the curve from time zero to infinity. In addition, in the light of the reports of Guerriero et al. [27] and Delker et al. [28], indicating that there is a short rapid washout phase with elimination of radioactivity from the kidneys, chiefly affecting the first hours of the time activity curve but with a small influence present after 8 h, we also calculated (fc_0-8_) the fractional contribution of the first 8 h to the total area under the curve. Examples of all these fractional contributions are shown on a typical curve for the kidney in Fig. 1.

### Simplification of the measurements

As a standard, absorbed dose (AD_147_) and *t*_eff_ (*t*_eff147_) are calculated using single exponential fit to data from 3 measurements 1, 4, and 7 days after start of the therapy. As a first attempt to simplify the dosimetry measurements, two calculations of the absorbed dose (AD_14_ and AD_17_) and *t*_eff_ (*t*_eff14_ and *t*_eff17_) were performed, using the single exponential functions crossing only two points (1 and 4 days, and 1 and 7 days, respectively). In addition, absorbed doses were calculated using a single measurement (at 4 days), assuming the median of our 777 patients *t*_eff_ 52 h (AD_4/52_) and using Eq. 7 (adapted to activity concentration) in the paper by Hänscheid et al. [29] (AD_4/H_). Since the standard method in the paper by Hänscheid et al. [29] is based on 2D measurements ending at day 4, we also performed a comparison between AD_14_ versus AD_4/52_ and AD_4/H_, based on the 3D measurements from our cohort of 777 patients.

### Statistical methods

Bland-Altman analyses for absorbed dose and *t*_eff_ values were performed for the comparisons between the different simplified methods, detailed above, relative to those based on the three-point measurement. Bland-Altman analyses of absorbed dose values relative to those based on days 1 and 4 measurement were calculated for the one time point method. Median, minimum, and maximum of the results were also calculated.

## Results

### Fractional contributions

**Fig. 2:**
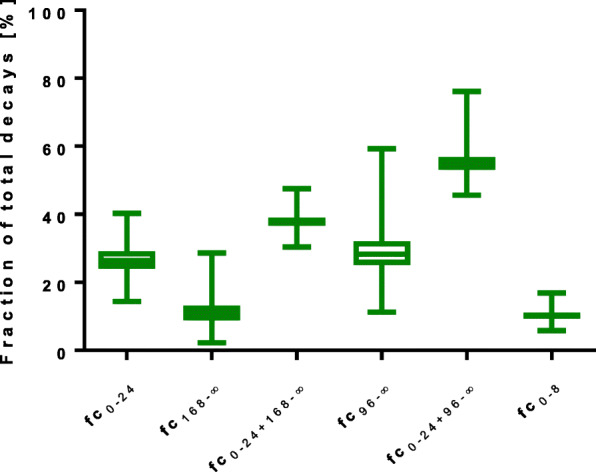
Fractional contribution of absorbed dose calculations for the left kidney to the total number of decays

The contributions to the absorbed dose to the left kidneys, as a fraction of the total absorbed dose from infusion start to infinity, are presented as a box-whiskers plot in Fig. 2. For the left kidneys, the extrapolation before the measurement at day 1 generally represented a little more than 25% of the absorbed dose, while the extrapolation after the measurement at day 7 contributed approximately 10%. This means that the total fractional contribution of extrapolations for the reference method was a little more than 35%. However, about 10% originates from the extrapolation during the first 8 h, during which the uptake and the 1st rapid elimination phase occur. The fractional contribution before day 1 and after day 4 was about 60%. For both the right and left kidneys, the fractional contributions from start to infinity are shown in Table 1. The difference in fractional contribution between the right and left kidney was generally small, even if they sometimes exceeded 20% in the individual patients. This small difference between the results in the right and left kidneys would not affect the conclusions.

**Table 1:** Fractional contribution for the right and the left kidney to the total absorbed dose for the time period from start to infinity

	Left kidney	Right kidney
fc_0-24_	26.4 (14.4–40.3)	26.1 (14.6–40.7)
fc_168-∞_	10.8 (2.2–28.6)	11.1 (2.5–29.7)
fc_0-24 + 168-∞_	37.8 (30.4–47.5)	37.8 (30.5–47.9)
fc_96-∞_	28.3 (11.2–59.3)	28.7 (12.1–61.8)
fc_0-24 + 96-∞_	55.0 (45.6–76.1)	55.2 (45.3–77.4)
fc_0-8_	10.2 (5.8–16.9)	10.0 (5.5–16.2)

Data are presented as median (range)

### Absorbed dose

**Fig. 3:**
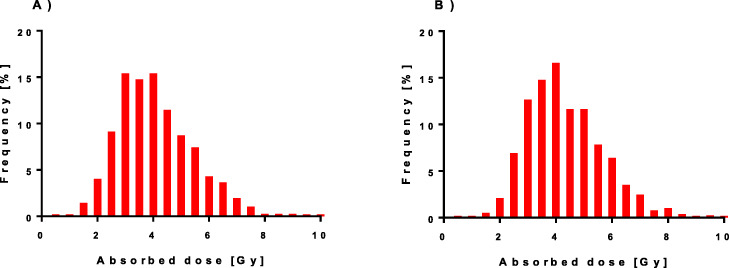
Histograms of the absorbed doses using our standard method AD_147,_ based on SPECT/CT measurements at days 1, 4, and 7, for the **a** left kidney and **b** right kidney

The absorbed dose to both the left and right kidneys, calculated with our standard method, is presented as histograms in Fig. 3. The results of the absorbed doses for the right and left kidneys are seen to be similar. During each 7.4 GBq PRRT cycle, the median absorbed doses were approximately 4 Gy, with a range from less than 1 Gy up to more than 10 Gy. The calculated median and range for all five methods are presented in Table 2.

**Table 2:** Absorbed doses for the different methods

Method	Left kidney	Right kidney
AD_147_	3.9 (0.6–9.8)	4.1 (0.6–12.6)
AD_14_	3.8 (0.5–10.3)	4.1 (0.6–12.3)
AD_17_	4.1 (0.6–10.2)	4.3 (0.6–13.1)
AD_4/52_	3.6 (0.5–9.0)	3.8 (0.6–11.8)
AD_4/H_	3.7 (0.5–9.4)	4.0 (0.6–12.0)

Data are presented as median (range) in Gy per 7.4 GBq

### Comparison of the simplified absorbed dose calculations

**Fig. 4:**
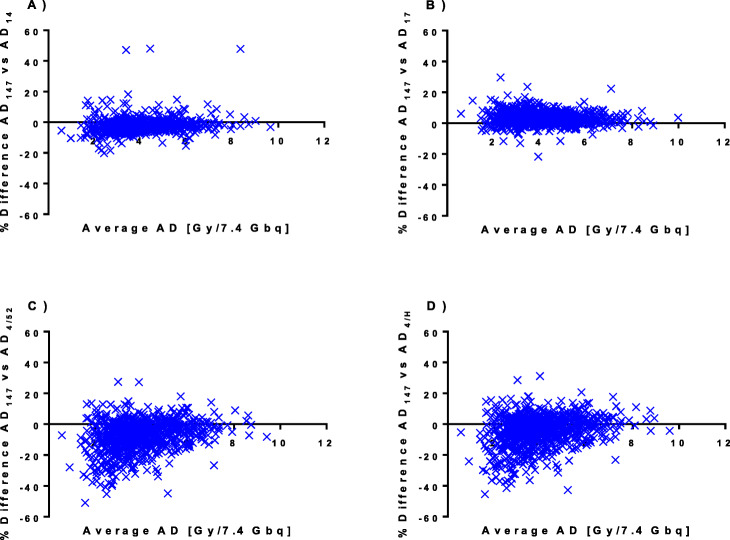
Bland-Altman plots of the percent difference versus the mean for the left kidneys in our standard method AD_147_ versus the simplified methods **a** AD_14_, **b** AD_17_, **c** AD_4/52_, and **d** AD_4/H_

Bland-Altman plots of the agreement between the simplified methods and our standard method are presented in Fig. 4. Differences between the simplified methods and our standard method are also summarized in Table 3 showing the median and range absorbed dose calculated by each method. The difference between methods was in general quite small (merely a few percent) although individual values varied considerably. For both AD_14_ and AD_17_, the differences were in general less than 20% but occasionally up to 50% and 30% for AD_14_ and AD_17_, respectively. For the methods employing only one measurement point (AD_4/52_ and AD_4/H_), the overestimation of the absorbed dose was generally less than 10% but was occasionally as high as 30% while the underestimations were greater, usually less than 20% but sometimes 40%, or even 50% in some cases.

**Table 3:** Percent difference of the simplified methods for absorbed dose calculations and the effective half-lives

		Left kidney	Right kidney
Percent difference in Absorbed dose	AD_147_ vs AD_14_	− 2.5 (− 20.1–71.8)	− 2.6 (− 39.6–52.4)
	AD_147_ vs AD_17_	2.9 (− 21.6–29.6)	2.8 (− 17.7–31.4)
	AD_147_ vs AD_4/52_	− 6.4 (− 51.0–27.4)	− 6.0 (− 59.2–27.3)
	AD_147_ vs AD_4/H_	− 3.3 (− 45.5–31.2)	− 3.1 (− 57.9–29.8)
	AD_14_ vs AD_4/52_	− 2.9 (− 67.5–4.5)	− 2.6 (− 63.3–4.8)
	AD_14_ vs AD_4/H_	0.0 (− 62.3–6.1)	0.4 (− 58.3–6.0)
Percent difference in effective half-lives	t_eff,147_ vs t_eff,14_	− 8.8 (− 79.1–89.4)	− 8.6 (− 87.6–66.0)
	t_eff,147_ vs t_eff,17_	0.0 (− 8.5–4.0)	0.0 (− 8.5–4.3)

Data are presented as median (range)

**Fig. 5:**
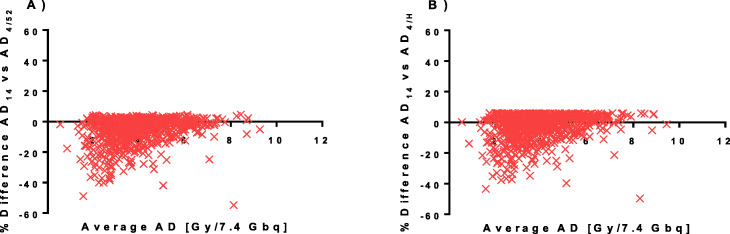
Bland-Altman plots of the percent difference versus the mean for the left kidneys in the simplified method AD_14_ versus the single time point methods **a** AD_4/52_ and **b** AD_4/H_

The single time point methods (AD_4/52_ and AD_4/H_) are compared to the method excluding the day 7 measurement (AD_14_) in the Bland-Altman plot in Fig. 5. Numerical values of the differences between AD_14_ versus AD_4/52_ and AD_4/H_ are presented as median and range in Table 3. The AD_4/52_ and AD_4/H_ did not overestimate the absorbed dose versus AD_14_ by more than a few percent (< 6%). However, the underestimates were occasionally 40% and in rare cases as much as 60%.

### Effective half-life

**Fig. 6:**
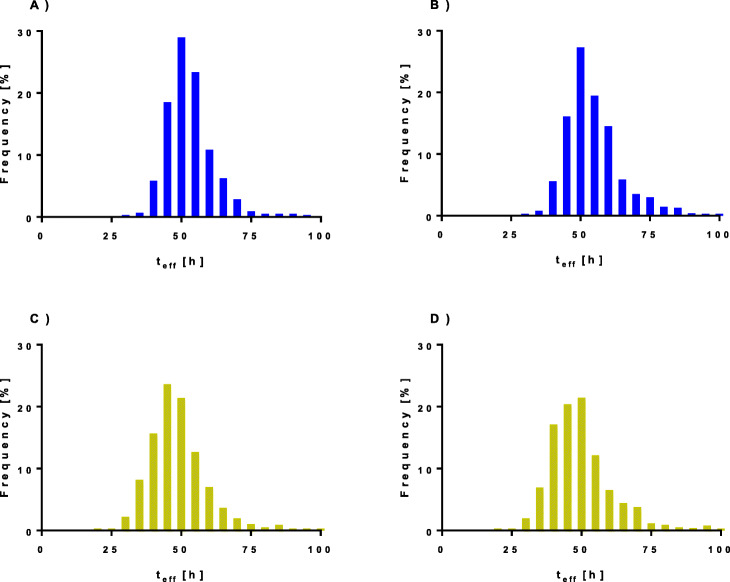
Histograms of the effective half-lives in the absorbed dose calculation using our standard method (AD_147_) for the **a** left kidney and **b** right kidney and using the simplified method AD_14_ for the **c** left kidney and **d** right kidney

Histograms of the estimated effective half-life *t*_eff147_, for the left and right kidneys, are presented in Fig. 6. A median value of 52 h was observed for both the left and right kidneys. In 90% of the patients, the *t*_eff147_ values ranged from 41 to 68 h for the left kidneys and 41 to 75 h for the right kidneys. However, in 5% of patients (both kidneys), *t*_eff147_ was lower than 41 h and occasionally as low as 30 h, and also, in 5% of the patients, *t*_eff147_ exceeded 68 h for the left kidney and 75 h for the right kidney, and sometimes reached almost 100 h.

The results of the *t*_eff17_ were almost the same as *t*_eff147_, differing less than 0.5 h in more than 90% of the kidneys. The only exception was the maximum value for the right kidney that in a single case exceeded 100 h.

The *t*_eff14_ showed a median of 48 h with a range from 20 h to the physical half-life of 6.7 days, and in 90% of the observed patients, the *t*_eff14_ ranged 34 to 69 h for the left kidneys and 35 to 73 h for the right kidneys.

### Comparison of the simplified effective half-life calculations

**Fig. 7:**
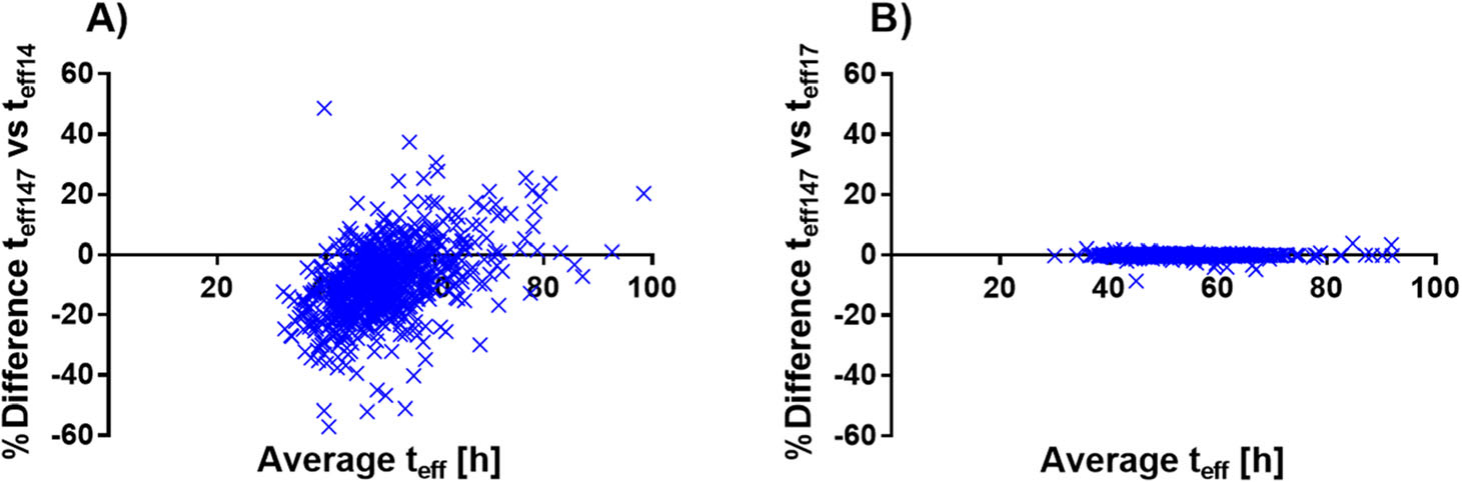
Bland-Altman plot of the effective half-life (*t*_eff_) for the reference method using data from 1, 4, and 7 days (*t*_eff147_) versus the simplified methods **a** using data from 1 and 4 days (*t*_eff14_) and **b** using data from 1 and 7 days (*t*_eff17_)

Figure 7 shows a Bland-Altman plot comparing *t*_eff147_ versus *t*_eff14_ and *t*_eff17_ for the left kidney. In Table 3, the numerical values for both left and right kidneys are presented as median (range).

For both the left and right kidney, the median difference between *t*_eff147_ and *t*_eff17_ was 0%, in more than 90% of the kidneys less than ± 0.5%, and all within ± 10%.

The median difference between *t*_eff147_ and *t*_eff14_ was − 9% for both the left and right kidneys, and in 90% of the patients, the difference ranged from − 27 to 11% and − 29 to 14% for the left and right kidneys, respectively. However, in 5% of the patients, the difference between *t*_eff147_ and *t*_eff14_ was larger and ranged from − 27 to − 79% and − 29 to − 88% for the left and right kidneys, respectively. In 5% of the patients, these differences were larger still and ranged from 10 to 89% and 14 to 66% for the left and right kidneys, respectively.

## Discussion

In this study, both extrapolations and simplifications in kidney dosimetry during ^177^Lu-DOTATATE therapy were analyzed. To ensure reliable results, extrapolations should be as small as possible and preferably not exceed 20% of the total decays in the volume [20]. In this study, the extrapolations for the time period following day 7 measurement were fairly low and present no problem in compliance with these criteria. This was not the case for the extrapolation before day 1 measurement, corresponding to approximately 25% of the total dose from time zero to infinity. Thus, it is of importance to consider the dose delivered in the early phase during the first hours. Even if the extrapolation between 8 h and 1 day represents more than 10%, this does generally not exceed 20%. Furthermore, reduction of the extrapolation time would involve SPECT/CT imaging during nighttime, which is not feasible in the clinical daily practice. The total approximately 30% extrapolation is not optimal but challenging to reduce. Further investigations regarding the effect of early time point measurements on the absorbed dose calculation are warranted. The objective is to simplify the dosimetry procedures as much as possible, mainly by reducing the number of measurements, without jeopardizing the accuracy of the absorbed dose calculations. Hänscheid et al. [29] in their paper concluded that the absorbed dose can be deduced with reasonable accuracy from a single measurement 4 days after the administration. They also concluded that deviations from the monoexponential function may introduce additional errors. Their study cohort consisted of 29 patients who underwent scintigraphy by planar imaging up to 4 days after activity administration. In the present study, we analyzed our data from 777 patients who had undergone SPECT/CT imaging at three time points at 1, 4, and 7 days following ^177^Lu-DOTATE infusion and compared these results with those based on measurements at a single time point, according to Hänscheid et al. For a more thorough assessment of the differences between dosimetry protocols, we compared the proposed single time point method not only to our standard three time point protocol, AD_147,_ but also to one that excludes the last time point, AD_14_. This resulted in an extrapolation after day 4 measurement point of about 30% and a total extrapolation of about 50% for the AD_14_ method, which is considerably more than the accepted 20%. Consequently, with a simplified absorbed dose calculation, it is crucial to determine the extrapolations in the reference method because the effect of using a reference with large extrapolations will be several unaccounted uncertainties. The absorbed doses in kidneys in our study were in good agreement with those reported by others [30]. All the tested methods for kidney dosimetry produced a median difference of 5.2% or less, an entirely acceptable difference in absorbed dose calculations in PRRT. However, the problem is that since PRRT is a radiation treatment, a good agreement on average is not sufficient to determine whether the accuracy is good enough. Thus, the absorbed dose calculated for an individual patient must yield the same result independent of the method applied. To comply with this requirement, the range of differences between the methods must be low. With our standard method, AD_147_ and the AD_17_ protocol, the differences were generally less than 20%, but in occasional cases nearly 30%. Although this may be considered sufficient, one must question if it is indeed good enough in the specific context of targeted radiotherapy whereby the amount of administered activity is tailored for the individual patient [8, 31].

Further, if the *t*_eff_ will be used to perform dosimetry based on one SPECT/CT at 24 h for subsequent treatments, according to the method of Garske et al. [31], then, the accuracy of *t*_eff_ is also highly important to avoid increased errors in the absorbed dose estimations in the subsequent treatment cycles. As expected, *t*_eff17_ agreed well with *t*_eff147,_ indicating that simplification of kidney dosimetry by means of a two time points (1 and 7 days) SPECT/CT protocol is feasible. The differences in absorbed doses between the AD_14_ and AD_147_ protocols were in general less than 20%. However, in individual patients, this difference was 30% and occasionally as high as 50% for the left kidneys and 70% for the right kidneys. The differences in *t*_eff_ between protocols was much larger and was as high as ± 90% when *t*_eff147_ and *t*_eff14_ was compared, making the latter protocol unreliable. The differences between the two single time point methods (AD_4/52_ and AD_4/H_) versus AD_147_ were found too high to be recommended, according to the calculations based on our data in the present patient cohort. Because this contradicted the results of an earlier study [29], we further investigated the difference between these one-point methods (AD_4/52_ and AD_4/H_) and the simplified AD_14_ method, based on SPECT/CT at two time points. It was particularly interesting to note that the AD_4/52_ and AD_4/H_ protocols never overestimated the absorbed dose by more than approximately 6%, as compared to the AD_14_ method. The underestimation for AD_4/52_ and AD_4/H_ was generally less than 30%, which may explain the favorable results reported in a relatively small sample of patients using planar imaging [29]. However, with less extrapolation, SPECT/CT measurements, and the considerably larger number of patients in the present study, these methods yielded too uncertain results. There may be several reasons for our deviating results, as compared to those reported previously [29]. It is reasonable that data from measurements at only one time point will introduce uncertainties in the calculations. Another factor, pointed out by Hänscheid et al. [29], is that deviations from the monoexponential decay in the slow elimination phase from the kidneys may be encountered. Notably, no evaluation of the uncertainties for these data was performed, since much data needed on the early measurements were not available. Thus, the results of the method comparisons need to be further assessed including an analysis of the uncertainties of the methods. Further, the true value of *t*_eff_ may in some patients be below 29 h or above 96 h. Probably, also other factors may impact the calculations. The uptake kinetics before the day 1 measurement is hitherto less studied and needs to be further explored to improve our knowledge regarding this early phase/phases.

## Conclusion

When simplifying the dosimetry protocol for estimation of the absorbed dose to the kidneys, it was of the uttermost importance to incorporate a calculation of the fractional contribution for the extrapolations included in the reference method. Calculations of the absorbed dose based on measurements at only one time point were unreliable. Measurements at an early and a late time point produce more reliable results, and an intermediate measurement is preferable to get an idea of the goodness of the fit.

## Data Availability

The datasets used and/or analyzed during the current study are available from the corresponding author on reasonable request.

## Abbreviations

^177^Lu-DOTATATE: ^177^Lu-DOTA-D-Phe1-Tyr3-octreotate
ACDF: Activity concentration dose factor
AD: Absorbed dose
BED: Biological effective dose
DF: Dose factor
MEGP: Medium energy general purpose
NET: Neuroendocrine tumor
OSEM: Ordered subset expectation maximization
PRRT: Peptide receptor radionuclide therapy
*t*_eff_: Effective half-life
VOI: Volume of interest
